# A Canonical Model of Multistability and Scale-Invariance in Biological Systems

**DOI:** 10.1371/journal.pcbi.1002634

**Published:** 2012-08-09

**Authors:** Frank Freyer, James A. Roberts, Petra Ritter, Michael Breakspear

**Affiliations:** 1 Bernstein Focus State Dependencies of Learning & Bernstein Center for Computational Neuroscience, Berlin, Germany; 2 Department Neurology, Charité - University Medicine, Berlin, Germany; 3 Division of Mental Health Research, Queensland Institute of Medical Research, Brisbane, Queensland, Australia; 4 Max Planck Institute for Human Cognitive and Brain Sciences, Leipzig, Germany; 5 Berlin School of Mind and Brain & Mind and Brain Institute, Humboldt University, Berlin, Germany; 6 School of Psychiatry, University of New South Wales and The Black Dog Institute, Sydney, New South Wales, Australia; 7 The Royal Brisbane and Woman's Hospital, Brisbane, Queensland, Australia; University of Oxford, United Kingdom

## Abstract

Multistability and scale-invariant fluctuations occur in a wide variety of biological organisms from bacteria to humans as well as financial, chemical and complex physical systems. Multistability refers to noise driven switches between multiple weakly stable states. Scale-invariant fluctuations arise when there is an approximately constant ratio between the mean and standard deviation of a system's fluctuations. Both are an important property of human perception, movement, decision making and computation and they occur together in the human alpha rhythm, imparting it with complex dynamical behavior. Here, we elucidate their fundamental dynamical mechanisms in a canonical model of nonlinear bifurcations under stochastic fluctuations. We find that the co-occurrence of multistability and scale-invariant fluctuations mandates two important dynamical properties: Multistability arises in the presence of a subcritical Hopf bifurcation, which generates co-existing attractors, whilst the introduction of multiplicative (state-dependent) noise ensures that as the system jumps between these attractors, fluctuations remain in constant proportion to their mean and their temporal statistics become long-tailed. The simple algebraic construction of this model affords a systematic analysis of the contribution of stochastic and nonlinear processes to cortical rhythms, complementing a recently proposed biophysical model. Similar dynamics also occur in a kinetic model of gene regulation, suggesting universality across a broad class of biological phenomena.

## Author Summary

Biological systems are able to adapt to rapidly and widely changing environments. Many biological organisms employ two distinct mechanisms that improve their survival in these circumstances: Firstly they exhibit rapid, qualitative changes in their internal dynamics; secondly they possess the ability to respond to change that is not absolute, but scales in proportion to the underlying intensity of the environment. In this paper, we study a simple class of noisy, dynamical systems that mathematically represent a very broad range of more complex models. We hence show how a combination of nonlinear instabilities and state-dependent noise in this model is able to unify these two apparently distinct biological phenomena. To illustrate its unifying potential, this simple model is applied to two very distinct biological processes – the spontaneous activity of the human cortex (i.e. when subjects are at rest), and genetic regulation in a bacteriophage. We also provide proof of principle that our model can be inverted from empirical data, allowing estimation of the parameters that express the nonlinear and stochastic influences at play in the underlying system.

## Introduction

Biological systems are optimized to survive in environments whose properties may vary greatly, such as changes in the biochemical environment of bacteria across several orders of magnitude, or even qualitatively, such as seasonal variations that banish food sources and prohibit foraging behavior in some mammalian species. Multistable dynamics and scale-invariant fluctuations are two complex dynamical processes whose presence in a wide variety of biological organisms suggests an adaptive role where they occur. The former enables switching amongst a wide variety of dynamical scenarios, whereas the latter ensures sensitivity to environmental fluctuations even if their background ambient intensity scales across several orders of magnitude. Their co-existence would allow a system to express two (or more) fundamentally distinct dynamical behaviors whilst maintaining scale-invariant fluctuations within and between each of these. The objective of this paper is to elucidate the basic dynamical mechanisms of these two phenomena and show how they can be studied within a unifying framework. We take the human alpha rhythm, which exhibits both multistability and scale invariant fluctuations [1], as a paradigmatic example and show how a recently proposed biophysical mechanism [2] is a specific example of the present, more general dynamical framework. We also investigate multistable dynamics in a kinetic model of gene regulation [3].

Mathematically, multistability corresponds to the presence of multiple concurrent state-space attractors, each with their own basin of attraction. System noise is required to erratically knock the system's state vector from attractor to attractor (for review, see [4], [5]. A classic example in the human perceptual system is binocular rivalry, the abrupt alternations between two discrete percepts that occur when different images are presented to each eye [6], [7]. Multistability is also found in the human motor system, for example when paced finger tapping switches between anti-syncopation and syncopation [8], [9]. In the setting of perceptual decision-making, multistability between both possible choices is thought to arise just before the outcome of a two alternative choice task [10], [11], [12]. Multistability has been reported in a wide variety of other biological contexts - including the cellular mechanisms of working memory [13], and gene expression, where it underlies cellular differentiation [14] and epigenetic variability in genetically identical cell lines [15]. These observations motivate the search for generic mechanisms not limited to a specific model or context.

In the framework of dynamical systems, multistability corresponds to the exploration of a multi-attractor landscape under the influence of system noise. Although the nature of the dynamical landscape has been extensively mapped, system noise is almost invariably introduced in biological contexts as an additive stochastic term. This contrasts with the treatment of stochastic effects in econometrics, where a complex relationship between trade volume (system activity) and volatility (system stochasticity) is a well-known property of financial systems [16]. This more complex, state-dependent relationship is also observed in a wide variety of biological organisms including bacterial chemotaxis [17] as well as complex physical systems. State-dependent fluctuations are arguably a defining feature of human cognition, being present in perception (the “Weber-Fechner law”; [18]), movement (“Fitt's law”; [19]) and even computation (“Hick's law”) where such operations appear to underlie nonverbal numerical processing in humans and monkeys [20], [21] and infants [22]. The almost ubiquitous observation of “state-dependent computations” [23] challenges the approach of simply adding system noise to dynamical states in computational models of the brain. Hence, state-dependent fluctuations and multistability are both present in the perceptual, cognitive and motor systems of the brain, ostensibly allowing the brain to adopt distinct functional modes, whilst ensuring uncertainty can be represented adaptively within and between these modes. For example, perceptual switching during binocular rivalry between visual stimuli differing by an order of magnitude along at least one physical dimension (such as contrast) would necessitate tight coupling of bistability and scale-invariant fluctuations in visual cortex in order to ensure equivalent perceptual representations across transitions. More generally, consider a distributed perceptual system that encodes its beliefs about hidden sensory causes in its mean state, whilst the precision of those beliefs is encoded in the dispersion of those states (see for example [24]). Accordingly, in the presence of perceptual ambiguity, the co-existence of state-dependent fluctuations and multistability would allow such a system to switch between several competing perceptual representations whilst keeping the precision of those beliefs relatively constant. Without this coupling, beliefs regarding the more intense aspects of the external environment would inevitably be held with greater precision regardless of their veracity.

Spontaneous activity of the human cortex is dominated by high amplitude 10 Hz oscillations, strongest over the posterior cortex - the so-called alpha rhythm. Knowledge of the human alpha rhythm dates back to the earliest recordings of electro-cortical activity by Hans Berger in 1924, yet its mechanisms remain poorly understood. In contrast to the widely held belief that the human alpha rhythm continuously “waxes and wanes”, it rather bursts erratically between two distinct ‘modes of activity’ [1]. Temporal fluctuations of power in each of these modes are not constant, but rather scale in proportion to the mean power of the modes. Spontaneous activity of the human cortex hence exhibits clear evidence of both multistability and scale-free invariance. A biophysical mechanism for these key features of the human alpha rhythm was recently established in a model of large-scale brain activity [2]. This neural field model describes the large-scale dynamics of corticothalmic activity, constrained by key neurophysiological properties [25]. When endowed with appropriate biophysical properties, this model showed a remarkable concordance with the multistable properties of the human alpha rhythm. A crucial process underlying this convergence between theory and experiment was the state-dependent gating of stochastic inputs to the specific thalamic nucleus (the key relay centre of the brain) by oscillatory feedback from the cortex. Proximity of voltage-dependent NMDA channels to ligand-gated ion channels was proposed to underlie this key “state dependent” innovation [2], [26].

The biophysical model employed by Freyer et al. [2] has been validated across a wide range of states of arousal hence positioning this finding within a broad and unifying account of cortical activity [27]. However, whilst Freyer et al. [2] add an explanation of the alpha rhythm to this framework, they do not elucidate the deeper dynamical mechanisms at play, or whether they could be achieved by other model innovations. The objective of the present study is to address this in a simple algebraic (“normal form”) model of multistable oscillations and discuss the broader implications for other complex biological systems by demonstrating the same phenomena in a modulatory genetic network.

## Results

### Multistability and scale-free fluctuations in empirically recorded alpha activity

To provide an orientation for the present purposes, the quantitative properties of resting state, eyes closed EEG data are briefly re-iterated (see Materials and Methods for data acquisition and analysis). In addition to exemplifying the properties of multistability and scale-invariant fluctuations, analyses of all subsequent modeled systems follow the same principles as those employed here.

**Figure 1:**
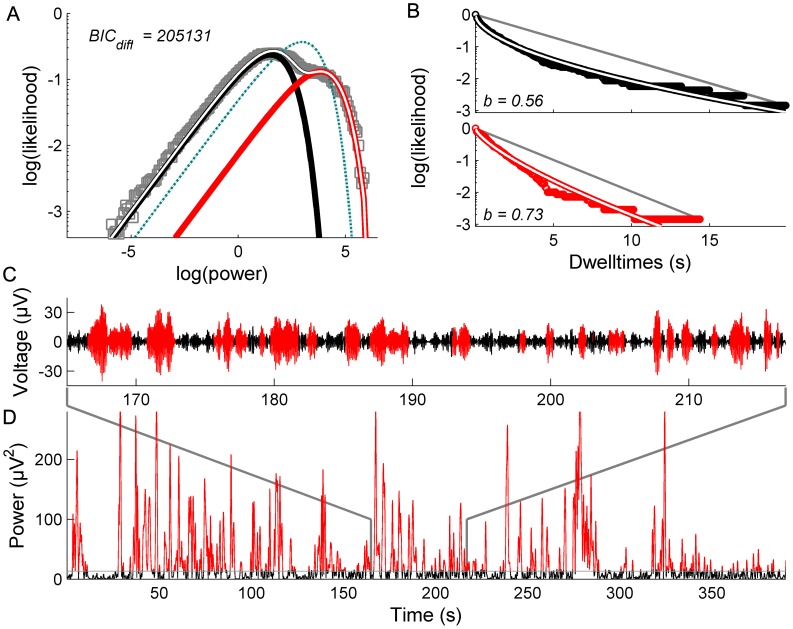
Multistability in human EEG data. A: PDFs derived from long recordings of EEG time series exhibit a low (black) and a high (red) amplitude mode. The overall PDF is well described by their bimodal sum (white). The superiority of the bimodal over the unimodal fit is reflected in the difference of the BIC values (*BIC_diff_*) B: The corresponding dwell times of each mode are well described by stretched exponentials (white). *b* indicates the dwell time stretched exponential exponent. The gray line indicates a simple exponential form. C: Time series of filtered (8–12 Hz) EEG. D: corresponding power fluctuations of 10 Hz oscillations (color coded according to the crossing of the distributions in panel A).

Fluctuations in power of 10 Hz oscillations burst erratically between a low amplitude mode and highly variable large amplitude excursions, which range across several orders of magnitude (Figure 1c,d). When viewed in double logarithmic coordinates, the PDFs derived from these time series exhibit clear bimodality (Figure 1a). Each of these modes can be well described with a simple exponential PDF of the form 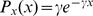 where *x* is the power and *γ* is the corresponding shape parameter. The overall time series is closely described by their sum. To formally compare the bimodal to the unimodal fit, we compare their Bayesian information criterion (BIC) values. The better model will yield lower BIC values, reflecting small residual variances after penalization for the number of free parameters. Crucially, although the peaks of these modes differ by several orders of magnitude, when viewed in these double logarithmic coordinates their relative widths are almost identical: This shows that their standard deviation (SD) scales in proportion to their mean.

The “dwell time” statistics further characterize the properties of these fluctuations [28]. These are defined as the successive durations that the system resides in each of the two modes (the boundary between the modes is defined by the crossing of the exponentials) and can be understood by inspecting their cumulative distribution functions (CDFs). For switching driven by a classic stochastic process - a simple Poisson process - the dwell time distributions conform to a simple exponential form 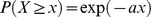 evident as a straight line with slope -*a* in semi-logarithmic coordinates (Figure 1b). In contrast, the empirical dwell time distributions in alpha fluctuations show evidence of long tailed statistics, which are well described by stretched exponentials of the form 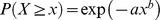. The parameter *b* captures the stretch, which imbues the dwell times with “fat right hand tails” and reflects the tendency of the system to be increasingly trapped in one state or another for 0<*b*<1. That is, the longer the system stays in one mode, the lower the likelihood of a switch to the other mode, in contrast to a Poisson process which has a constant failure rate.

### Bifurcations of a normal form model

As reviewed above, multistability occurs in dynamic systems when system noise causes the states to jump between two or more co-occurring phase-space attractors, each with their own basin of attraction [29]. While a number of dynamical scenarios with different sets of attractors can give rise to multistability [30], the multistable behavior of the alpha rhythm suggests a particular setting. Given that the human alpha rhythm jumps between a low amplitude mode and a high amplitude oscillatory waveform [31], bistability in this setting likely reflects switching between a fixed point and a limit cycle attractor [2]. In particular, whereas high amplitude alpha oscillations are strongly nonlinear, the low power fluctuations lack nonlinear structure [31], [32], arguing that the low amplitude mode more likely corresponds to fluctuations around a fixed point rather than a second limit cycle. Although the co-existence of these attractors can arise in a wide class of models describing complex systems, all are mathematically equivalent to the normal form of a Hopf bifurcation. A normal or canonical form is a set of reduced, approximating equations which are considered to preserve the essential features of the original system [33], [34]. Normal forms are usually analytically solvable and therefore allow a deeper insight into the geometric structure of the approximating equations [35]. Here we study the general normal form of a Hopf bifurcation, namely
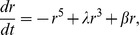
(1)

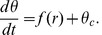
(2)(1) describes the amplitude dynamics with shape parameter λ and bifurcation parameter *β* (see below). (2) describes the dynamics of the system's phase. The function f(r) defines the relationship between amplitude and phase, i.e. the phase dynamics is amplitude dependent if 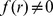. In the present paper we solely focus on the amplitude dynamics, and for reasons of simplicity we will only consider the case of a constant phase velocity, θ_c_ = 10 Hz with f(r) = 0. Note that (1) is an odd function so that whatever holds for r>0 also holds for r<0 (because the amplitude and its derivative are both inverted). Hence, without loss of generality we need only consider the case r≥0.

**Figure 2:**
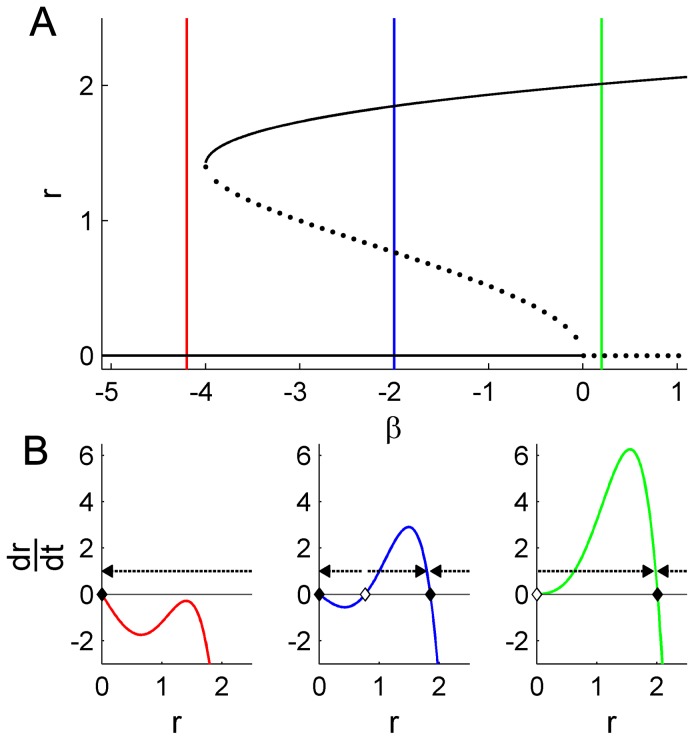
Varying *β* for a fixed *λ* = 4 yields a subcritical Hopf bifurcation. A: Bifurcation diagram for state parameter *β* showing the region of bistability. Attracting solutions shown in solid. The unstable repellor (or *seperatrix*) is shown as the dotted curve. The fixed point loses stability at *β* = 0. B: The geometry of equation (1) for three different values of *β*. Attractors are shown as solid diamonds and repellors as open diamonds. Bistability corresponds to the blue setting.

Because (1) is always zero for *r* = 0, the system always has a fixed point at the origin. This fixed point is an attractor if the RHS of (1) is negative in the immediate neighborhood of zero. Otherwise it is a repellor. If it is negative for all r>0 then the origin is the system's *global attractor*. However this fifth degree polynomial can have at least two positive roots and, due to the rotation introduced by (2), the system can hence have up to two limit cycles of finite positive amplitude *R*. Any such limit cycle will be an attractor if the sign of (1) is positive for *r*<*R* and negative for *r>R*. Otherwise it will be a repellor. In Figure 2a, we consider a variety of scenarios with the shape parameter fixed at *λ* = 4 and different values of the bifurcation parameter *β*. In the first case (red; 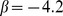 the RHS of (1) is strictly negative (leftward moving) for *r*>0 and hence the origin is the system's global attractor. However as *β* increases, the local maximum at r∼1.5 crosses zero at 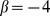, ensuring two limit cycles. The preceding geometric considerations imply that there is hence an unstable repellor (at 
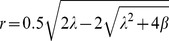
) and a stable attractor (at 
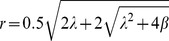
). An example (blue) is given for *β* = −2. With a further increase in *β*, the limit cycle repellor migrates inward and eventually (at *β* = 0) collides with the fixed point. Following this bifurcation (i.e. for *β*>0) geometry reveals that the fixed point is a repellor and the outer (and now only) limit cycle is the system's global attractor. Panel (b) shows the three possible roots of (1) plotted across a continuous range of *β* with locations of the three preceding scenarios indicated accordingly. This plot reveals the continuous unfolding of the three scenarios just outlined, with the abrupt discontinuities - heralding bifurcations - at *β* = −4 and *β* = 0. This is the canonical *sub-critical* Hopf bifurcation, so-called because limit cycle solutions occur below the loss of stability of the fixed point at *β* = 0. Generally, bistability is confined to the region 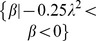 where the attracting fixed point and attracting limit cycles have basins of attraction separated by the unstable limit cycle (hence also called a *seperatrix* or *basin boundary*).

**Figure 3:**
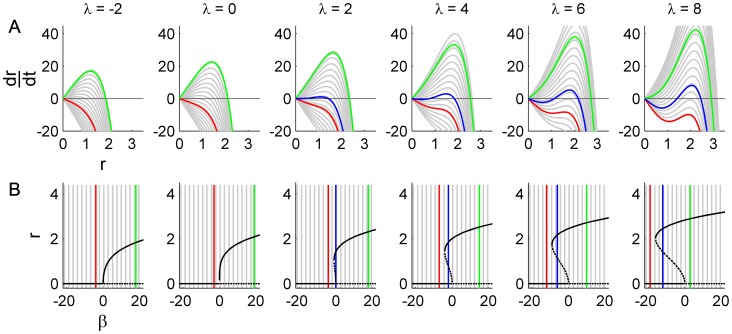
Complete family of Hopf bifurcations. A: The geometry of equation (1) for different values of the shape parameter *λ*. B: Corresponding roots of equation (1) (solid: stable; dashed: unstable). As the shape parameter *λ* increases, the bifurcations morph from supercritical (left) to subcritical (right). Exemplary values of bifurcation parameter *β* that yield different attractor landscapes are indicated in red (fixed point), blue (coexisting unstable and stable limit cycles) and green (stable limit cycle).

Similar geometric considerations across a range of values of the shape parameter *λ* reveal all possible dynamic scenarios of this system (Figure 3). The limit cycle repellor - necessary for the subcritical Hopf bifurcation and hence bistability - occurs across a broad region of parameter space (one example in blue) and can hence be considered a *structurally stable* solution to the system. However, for *λ*<0, the quintic polynomial (1) has at most two zero crossings, i.e. two attractors. For *β*<0 the only attractor is at the origin *r* = 0, and as *β* increases through *β* = 0 (far left panels, red to green), this fixed point loses stability in favor of a single limit cycle attractor that grows continuously in amplitude from the origin. The absence of a point of inflexion for these values of *λ* precludes the possibility of the unstable limit cycle and hence bistability. Because limit cycle solutions occur strictly *above* the loss of stability of the fixed point at *β* = 0, this scenario corresponds to a *supercritical* Hopf bifurcation.

This system hence captures the complete family of Hopf bifurcations with a continuous transition between the super- and subcritical cases. A third order (cubic) polynomial is sufficient to model either a super-critical *or* a subcritical Hopf bifurcation, but not both (i.e. there is no single cubic function that allows a smooth transition between the two). The fifth order term can be thought of as a higher order “correction term” that allows one to model a physical system that could, in principle, express either a subcritical or a supercritical bifurcation depending on the smooth tuning of a control parameter (see [33]). This model hence provides a single computational platform to explore our primary hypothesis as well as any putative alternative hypotheses.

### Multistability in the normal form model

#### Switching with purely additive noise

**Figure 4:**
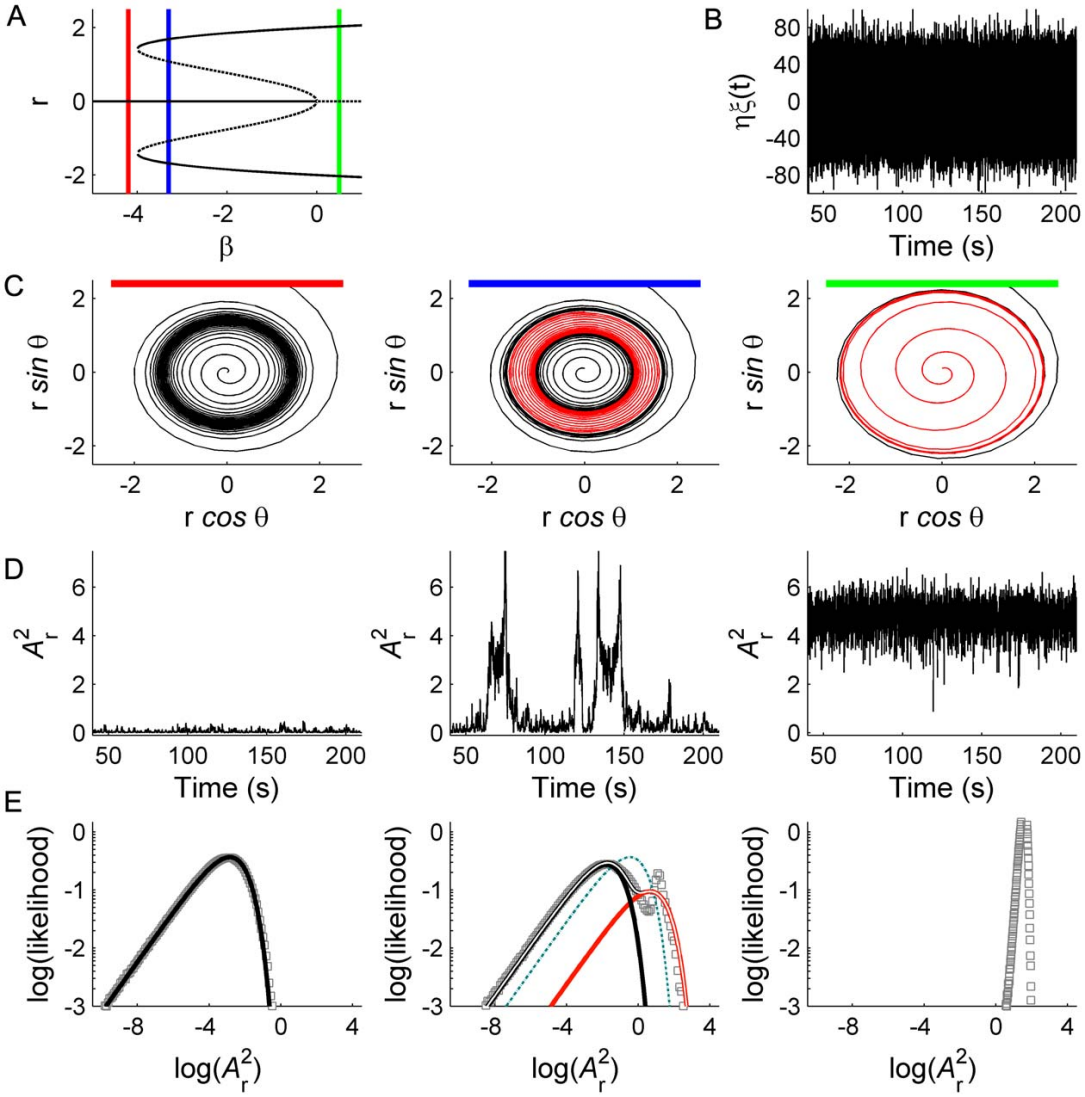
Bistability with purely additive noise for three different values of the bifurcation parameter *β*. A: Bifurcation diagram for *λ* = 4. The three values of *β* used here are indicated in red, blue and green. B: Time course of uncorrelated noise input 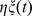. C: Phase portraits of example integrations. Black/red indicates inward/outward flow. D: Time series of example integrations. E: Corresponding power PDFs. 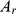 is the discrete-time analytic signal of *r* (i.e. the amplitude envelope of *r*).

Bistability in the subcritical setting represents the simplest likely case where the presence of noise may cause the bimodal switching observed experimentally. We hence study the behavior of this system in the presence of noise, introduced through the addition of a stochastic term to the RHS of (1),
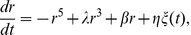
(3) Where 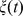 is an independently generated zero mean stochastic process with unit variance 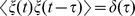 and 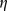 is a constant scaling coefficient. This *Langevin equation* has the classic form – purely deterministic terms plus a scaled state-independent noise term - employed widely in computational biology (for review, see [36]) and numerous other fields [29]. The noise term incorporates a wide range of potential stochastic influences occurring across a broad range of biological processes, such as thermal fluctuations or neuronal inputs from brain regions not explicitly modeled. Figure 4 illustrates the corresponding behavior of the three scenarios outlined in Figure 2. As expected, for sufficient noise amplitude (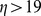) switching between low and high amplitude modes occurs exclusively in the region of bistability (blue; middle column). However, the corresponding bimodal PDF exhibits an obvious deviation from the empirical data (compare Figure 4e with Figure 1a). Whilst the mean increases by two orders of magnitude from the low to the high amplitude fluctuations, the standard deviation is constant. Hence in these double logarithmic coordinates, the high power mode is clearly thinner than its low power counterpart. A theoretical exponential PDF with the empirically motivated proportional scaling between mean and standard deviation (shown in red) is thus a poor fit to the second mode in these numerical data. The progressive thinning of the standard deviation with the increasing mean is also evident when comparing the difference between the PDFs of the unimodal fixed point (red; left column) and unimodal limit cycle (green; right column) solutions. Naturally the standard deviations in all three cases are of equivalent size because the fluctuations arise from the same term. The PDFs only appear thinner when viewed in logarithmic coordinates.

#### The role of multiplicative (state dependent) noise

In order for the standard deviation to scale with the mean, a state dependent noise term is clearly mandated. We hence introduce a first order multiplicative term via,
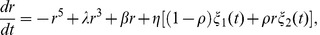
(4) Where 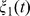 and 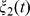 are uncorrelated and independent zero mean stochastic variables with unit variance, and 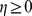 scales the overall noise influence. The constant 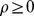 determines the relative influence of the state-dependent (multiplicative) noise term 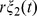 such that it effectively competes against the additive term 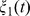. Note that if the stochastic influences are purely multiplicative (i.e. 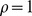) then the total stochastic influence tends to zero if the system approaches the fixed point 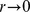. This leads to a rapid and permanent “quenching” of the system's activity and is thus of no further interest. Setting 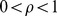 prevents this, whilst also allowing the multiplicative term to play an increasingly dominant influence away from the origin.


**Figure 5:**
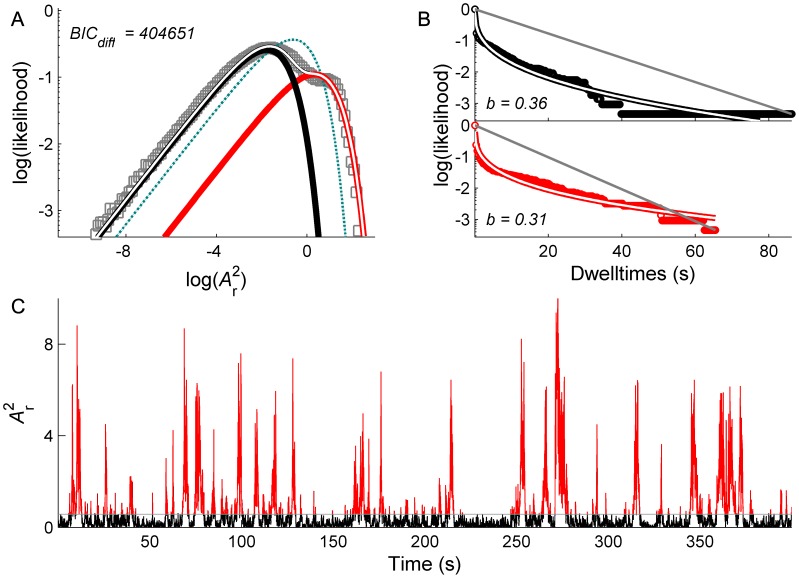
Multistability and scale-free fluctuations in the model with both additive and multiplicative noise. A: Bimodal power PDFs B: Stretched exponential dwell time CDFs for the two modes. C: Time series, showing transitions between low amplitude (black) and high amplitude (red) modes in the model system described by equation (4) with noise parameters η = 45, ρ = 0.61. For additional details please refer to legend of Fig. 1.

As shown in Figure 5, this system exhibits very close agreement with the bistable properties of human EEG (cf. Figure 1). In contrast to Figure 4, it is clear that the standard deviation now scales in proportion to the mean and, additionally, the dwell time distributions follow a stretched exponential form. This also affords insight into the origin of the trapping (long right hand tails) in the dwell time distributions: When the system first switches crosses the seperatrix into the low power mode, the noise terms exert a relatively strong influence, implying that a switch back across the seperatrix (into the high power mode) is reasonably likely. However, if by chance the system does not cross but instead approaches zero, the state-dependent stochastic term diminishes in influence and the system is relatively less likely to be perturbed back into the high power mode. Hence switching likelihood diminishes smoothly with dwell time (survival). Conversely, when the system crosses the separatrix into the right hand mode, the state-dependent term increases in effective strength, driving the system towards the erratic high amplitude fluctuations visible in the time series and trapping the system in the high power mode.

### Parametric analysis of noise and stability parameters

Whilst these basic processes are present in most human EEG recordings, there exists considerable inter-subject variability in the degree of bimodality, the relative height of the two modes and the stretch of the dwell time distributions ([1]; see supplementary Figure S1). For example, in a sample of 16 subjects the stretched exponential exponent *b* varied between 0.4 and 0.6 for the low power mode (mean 0.49) and from 0.5 to 0.9 for the higher mode (mean 0.68). Can these high-order statistics be used for subject-specific parameter estimation when inverting models of large-scale cortical activity? We exploit the simple parametric form of (4) by systematically varying the deterministic and stochastic terms and obtaining numerical time series, from which we estimate system statistics describing the extent of the bimodality and the shapes of the dwell time distributions for the two modes. This allows a proof of principle that the model parameters could be inferred from empirical time series data.

**Figure 6:**
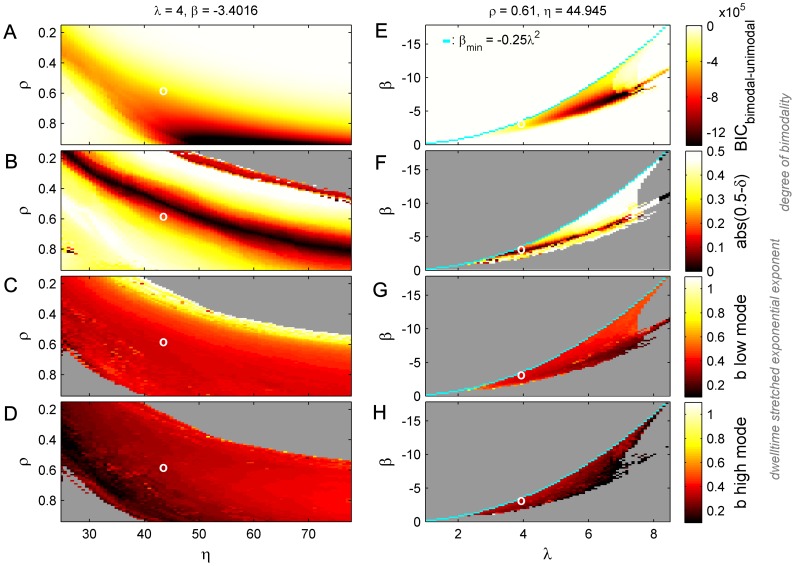
Summary system statistics for ‘degree of bimodality’. A,E: BIC difference between unimodal and bimodal fit; B,F: Relative height of the two modes (abs(0.5-δ)). Dwell-time stretched exponential exponent *b*, for low (C,G) and high (D,H) energy mode. Left column (panels A–D): System statistics for varying values of the noise parameters *η* and *ρ*, with fixed values for the shape parameter *λ* and bifurcation parameter *β*. Right column (panels E–H): System statistics for varying values of the shape parameter *λ* and bifurcation parameter *β*, with fixed values for the noise parameters *η* and *ρ*. White circles indicate the values of the parameters where they are fixed in the complementary panels. Cyan curve indicates theoretical minimum *β*-value for bistability, showing close agreement with numerical analysis.

The results for four summary statistics are shown in Figure 6, with attention restricted to regions in parameter space where bimodal activity arises. Results in Figure 6a–**d** show the impact of varying the two noise parameters ρ and η, while the model's shape and bifurcation parameters λ and β are varied in Figure 6**e–g**. Note that, consistent with Figure 3, bistability only exists in a thin strip of parameter space where 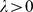. Four types of summary system statistics are shown. The top row depicts the relative goodness of fit of a bimodal compared to a unimodal fit, after penalizing for relative model complexity with Bayesian Information Criteria (BIC). This captures the depth of the bimodality - that is, the overall distinctiveness and separation of the modes (see [1]). The second row shows the relative height of the two modes and hence the ratio of the time that the system is trapped in either mode. The lower two rows show the degree of stretching of the dwell time distributions for the low and high power modes (third and bottom rows, respectively).

There are several noteworthy features of these plots: Firstly, the curves are well behaved (no smoothing has been applied) and are either monotonic in cross section, or unimodal. A specific experimentally derived estimate of any of the system statistics hence constrains the value of underlying system parameters to a relatively simple curve or loop (corresponding to contour lines) in each of these spaces. Secondly, although there do exist correlations between some of the parameters, they are sufficiently distinct that combining multiple system statistics generally further constrains the underlying parameter values. For example, the contours in Figure 6e for the BIC values are generally orthogonal to those in Figure 6**d** for the stretching of the low power mode. Hence specific values of each of these two system statistics will in general constrain the values of the model parameters *λ* and *β* to at most two possibilities (where the contour lines of each statistic cross).

Fixing the parameters and simulating the system multiple times yields an ensemble of time series from which can be extracted an ensemble of data features. Ensemble simulations show that each of the summary statistics (BIC difference, etc.) follow a Normal distribution (Supplementary Figure S1) whose mean corresponds to the value shown in Figure 6. This is crucial for formal model inversion since this allows a (pre)metric to be defined over these spaces, using a suitable function such as Kullback–Leibler divergence and hence estimates of the distance between parameter values. We also note that although the contour lines in these particular planes may not cross, we actually seek the intersection of the surfaces which yield these lines in cross-section and which more generally will have mutual intersections in the full dimensional space. This can be achieved with a suitable variational scheme such as [37].

**Figure 7:**
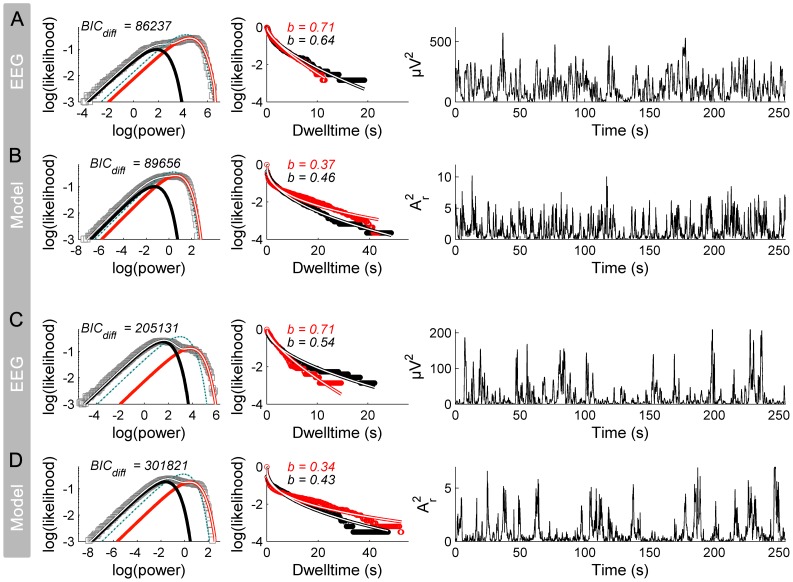
Illustration of two EEG data sets and corresponding model solutions. A: EEG data with frequent high amplitude excursions and short dwelltimes (i.e. less stretched dwelltime CDFs). B: corresponding model solution with *ρ* = 0.46, *η* = 30.4. C: EEG data with infrequent high amplitude excursions and long dwelltimes (i.e. more stretched dwelltime CDFs). D: corresponding model solution with *ρ* = 0.71, *η* = 57.6.

Although formal model inversion is beyond the scope of the present submission, two ‘proof-of-principle’ examples of model inversion from EEG data are shown in Figure 7. In the top row, system statistics of EEG data (Figure 7a) with frequent high amplitude excursions and short dwell-times (i.e. less stretched CDFs) are captured by the normal form model (Figure 7b) with noise parameters *ρ* = 0.46, *η* = 30.4, obtained from contours of Figure 6. In the bottom row, an empirical example with a smaller high power mode and longer dwelling (i.e. more stretched CDFs) is captured by increasing both the overall noise variance (to *η* = 57.6) and the ratio of multiplicative noise (to *ρ* = 0.71).

### Examining alternative hypotheses: Parameter noise and supercriticality

The notion that resting state cortex operates in the vicinity of a nonlinear instability is certainly not unique to the present contribution, although the majority of previous accounts have assumed that the bifurcation was supercritical (e.g. [31], [38], [39], [40], but see also [41], [42]). In this case, switching between a low and high amplitude mode arises from a “wandering” of the system across either side of the bifurcation threshold, corresponding to a random walk on the system's bifurcation parameter.

To investigate the dynamics arising in this scenario, we set the shape parameter lambda to -4 so that the system possesses a supercritical bifurcation, and endow the bifurcation parameter beta with mean reverting stochastic (Ornstein Uhlenbeck) fluctuations,
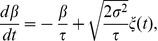
(5) where 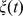 is an uncorrelated Wiener process. Beta will hence undertake stochastic excursions either side the bifurcation point, *β* = 0. The typical excursion duration and depth into either regime is determined by the correlation time τ and the variance σ. In particular, if τ diverges (i.e. 1/τ approaches zero), equation 5 becomes a Brownian walk on β. In this setting, the first return time for β (the dwell times for fluctuations on either side of the 0 axis) would show a power law distribution, which is contrary to the presence of stretched exponential dwell times in our EEG data. Decreasing τ (and hence introducing correlations) leads to an exponentially truncated first return time distribution (again not a stretched exponential). Should the system even yield a bimodal PDF, here we already encounter a major difference from the subcritical case, namely that the temporal statistics in the supercritical setting are determined by the (arbitrarily-imposed) characteristics of the noise through the correlation time parameter τ and do not emerge from the system's inherent dynamics.


**Figure 8:**
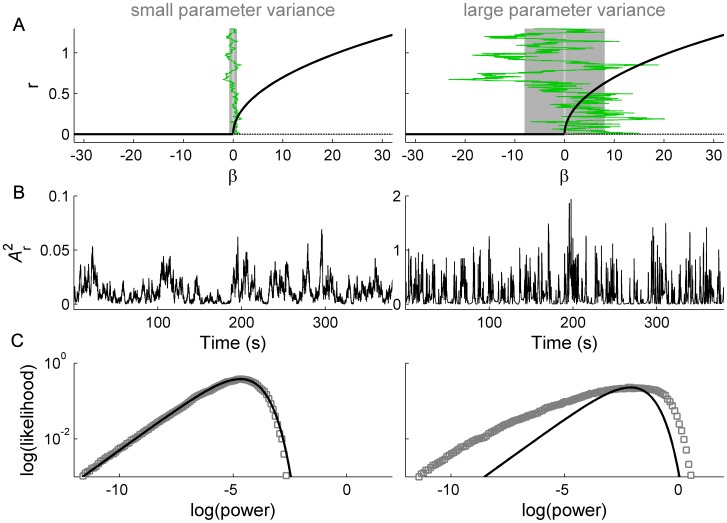
Two cases of parameter noise in the supercritical setting. Left column: Small noise variance, right column: Large noise variance. A: Supercritical Hopf bifurcation for *λ* = −4. The bifurcation parameter *β* is set to zero and perturbed at each integration time point with a mean reverting stochastic process, see equation (5). Examples of the resulting temporal excursions of *β* are depicted green. Grey boxes indicate standard deviations of the stochastic process. B: Resulting time-series of 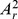. C: Corresponding PDF.

Two examples of this dynamic scenario are presented in Figure 8. For small to moderate parameter noise, power fluctuations express a unimodal exponential PDF, despite the emergence of occasional oscillatory activity in the time series. For moderate to large parameter noise, the system exhibits periods of high amplitude oscillatory behavior corresponding to parameter excursions well into supra-threshold territory. The probability distributions in these settings do not, however, show the clear bimodal distributions. Rather they generate broad, approximately unimodal forms that do not converge, even over long integration times towards any obvious simple probability distribution (multiple local minima are occasionally present in the PDFs). This is not particularly surprising since mean reverting stochastic (Ornstein Uhlenbeck) fluctuations are maximally (and unimodally) distributed around zero. In brief, the parameters of (5) play a crucial role in determining the data features, but impart an expected high penalty cost and generate a poor model fit. It hence seems unlikely that this scenario, with the increased complexity, can parsimoniously account for bimodal statistics in human EEG.

### Bistability in a biophysical model of corticothalamic activity

Having established the dynamical principles of bistability in a normal form model, we now revisit bistability in a detailed biophysical model of electrocortical activity (previously reported in [2]). This model describes local mean field dynamics of populations of excitatory and inhibitory neurons in the cortical gray matter as they interact with neurons in the specific and reticular nuclei of the thalamus [25]. These dynamics are governed by physiologically derived nonlinear evolution equations that incorporate synaptic and dendritic filtering, nonlinear firing responses, corticothalamic axonal delays and synaptic gains between presynaptic impulses and postsynaptic potentials. Resting state cortical activity has extensively been modeled by studying the noise-driven endogenous fluctuations. This yields a set of eight first-order stochastic delay differential equations. For a full model description including equations, please refer to [2], [25], [38].

**Figure 9:**
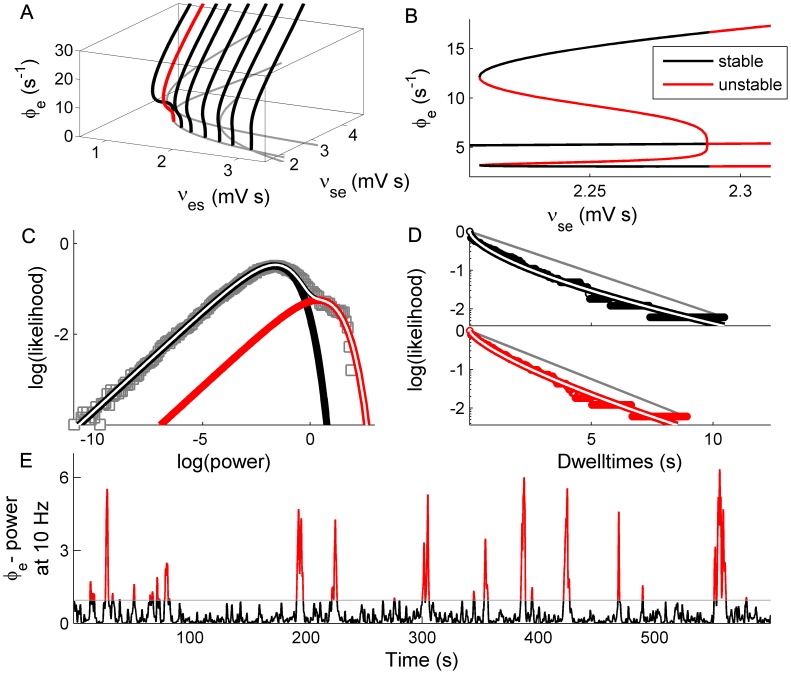
Scale-free bimodal fluctuations in a biophysical model of corticothalmic activity. Results are shown for fluctuating excitatory field potentials *φ_e_*. A: Full family of Hopf bifurcations identified by continuing solutions along a 10 Hz instability. B: Candidate sub-critical Hopf bifurcation (red curve in panel A). Stable attractors in black, unstable solutions in red. C: Candidate Power PDF with clear bimodal distribution. D: Corresponding stretched exponential dwell time CDFs. E: Power time series shows erratic switching.

Whereas in [2] examples of bistability were illustrated, we presently seek to more deeply explore the underlying bifurcation space and hence the core dynamical processes. We employ a numerical continuation scheme [43] to study the family of bifurcations occurring at the 10 Hz (alpha) instability within the subspace of this system's parameter space spanned by the excitatory connection strengths for the reciprocal feedback between cortex and thalamus, *ν_es_* and *ν_se_*. Although this delay differential system is substantially more complex than the preceding normal form, it nonetheless exhibits a comparable family of Hopf bifurcations with a continuous transition from sub- to supercriticality along a branch of 10 Hz instabilities (Figure 9a). We hence identify a candidate set of parameter values for a subcritical Hopf bifurcation (Figure 9b) and drive the system with a combination of additive and multiplicative stochastic inputs. In particular, following [44] we introduce a state-dependent feedback term from descending excitatory cortical input to the specific thalamic nuclei which modulates the strength of ascending stochastic input from subcortical sources (as motivated in [37]).

The model exhibits bimodal distributions of neural activity (Figure 9c), with long tailed dwell time distributions (Figure 9d), bearing strong resemblance to both the normal form model and empirical EEG. However, for other parameter combinations, the corticothalamic model also captures a broad range of other statistics of EEG, including epileptic seizures [27], [38], sleep spindles [27] and evoked potential waveforms [45] that do not occur in the simple normal form model.

### Bistability and multiplicative noise in a genetic regulatory network

Intriguingly, the processes we presently study in the human brain have been observed across a range of distinct different biological systems. For example, the crucial role of bistability has been recently reported in gene expression [14], [15], [46], [47] as has the existence of long-tailed distributions in gene expression [48]. State-dependent noise has also been reported to play a key role in bacterial chemotaxis [17]. Our analyses may thus speak broadly to other biological contexts.

We analyse a kinetic model of genetic regulation [3]. This model captures DNA-protein interaction in a small feedback network of genetic regulation in the temperate bacteriophage *λ* - a protoypical example of efficient phenotypic switching in response to an environmental signal [49], [50]. Please note that the bacteriophage λ is unrelated to shape parameter λ in equation (1). Briefly, the concentration of a single genetic repressor, *x*, initiates a sequence of DNA binding and dimerization at three sequential binding sites, each quickly reaching equilibrium due to its fast kinetic rates. The resulting DNA-protein complex then initiates transcription and production of *x* - hence enacting a positive feedback loop - which is offset by its degradation. Transcription also requires the presence of an RNA polymerase. Both transcription and degradation occur at slower time scales than binding and dimerization.

By assuming the fast kinetic equations are kept at (or close to) equilibrium in comparison to transcription and degradation, the evolution of *x* can be described by the differential equation,
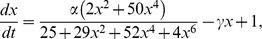
(6) where the dimensionless parameter *α* is a measure of the degree in which the transcription rate is increased above its basal rate by repressor binding, and *γ* is proportional to the relative strengths of the degradation and basal rates [3].


**Figure 10:**
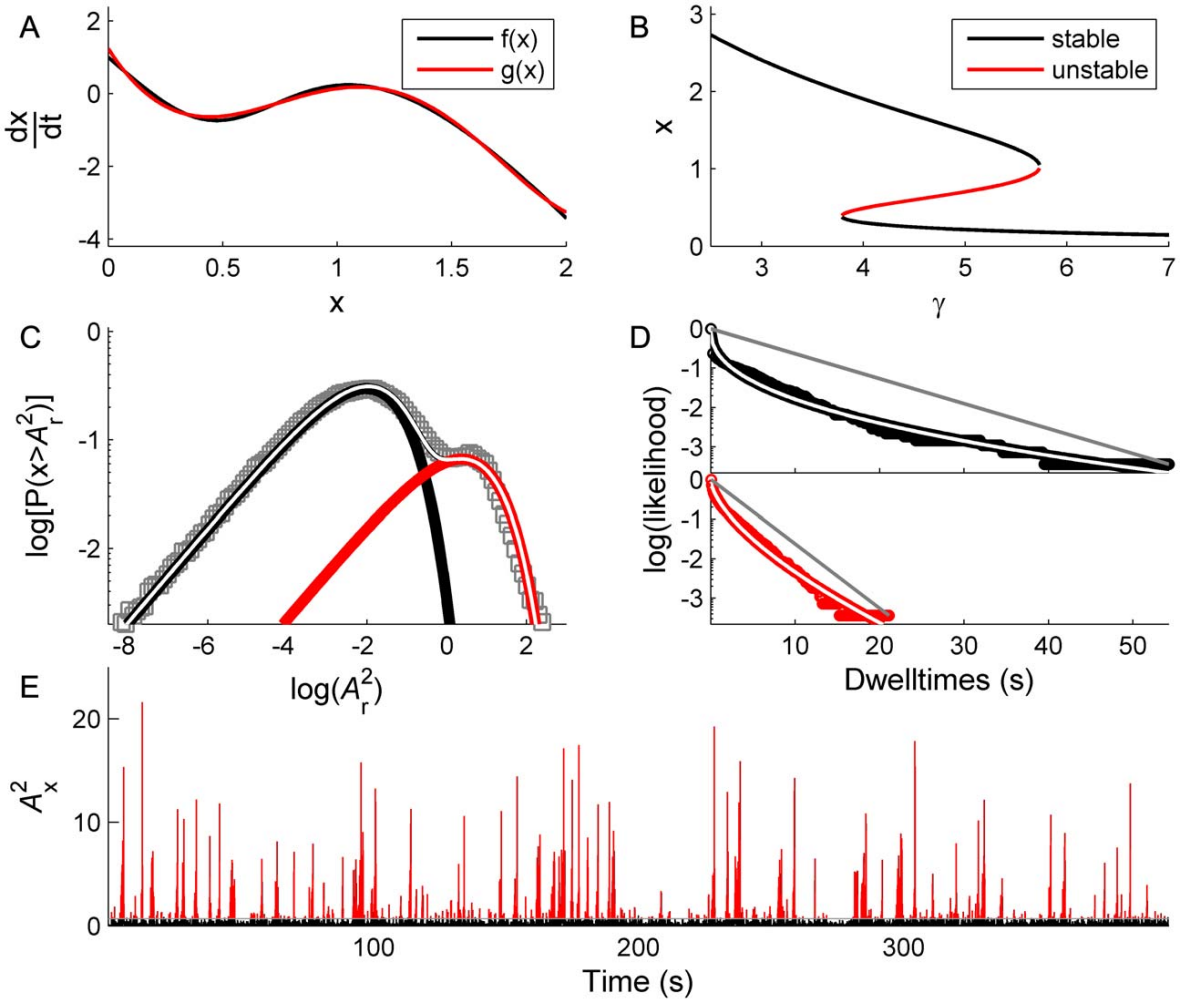
Bistability and trapping in a kinetic model of genetic regulation. A: Form of equation (6), *f(x)* (black), is well matched by best fitting quintic polynomial, *g(x)* (red). B: Bifurcation diagram of equation (6). C: Candidate Power PDF with clear bimodal distribution. D: Corresponding stretched exponential dwell time CDFs. E: Power time series shows erratic switching.

Despite the complex algebraic form of (6), it can be seen that for physiologically realistic values of *α* = 10 and *γ* = 5.5, this functional form has only one local maximum for *x*>0 and closely matches the qualitative shape of the best (least squares) fitting quintic polynomial (Figure 10a). Indeed, fixing the transcription rate parameter *α* = 10 and treating *γ* as a bifurcation parameter reveals a pair ofbifurcations yielding bistability between a high and low transcription rate occuring in the range 3.79<*γ*<5.73 (Figure 10b).

Stochastic influences in genetic networks are widely modelled, representing the action of thermal fluctuations, parameter variability, and the impact of more complex chemical processes also not explicitly modelled. Similarly, in large-scale neuronal systems stochastic inputs typically represent non-specific inputs from neuronal regions (such as sensory organs) not otherwise explicitly modelled. In both settings, in systems where noise interacts with a feedback loop it can be introduced via a state-dependent stochastic term. Figure 10c–e shows an example time series with a state-dependent noise term 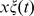 added to the RHS of (6). It hence exhibits a bursty time series for the amplitude distribution, with a bimodal PDF showing scale-free fluctuations, and long-tailed dwell time distributions, all in excellent agreement with the properties of the normal form model. Intriguingly, these bimodal, scale invariant distributions appear to replicate those observed in other genetic regulatory systems, such as Figure 7 of [41].

## Discussion

Detailed biophysical models - derived and parameterized using prior biological knowledge - are useful for elucidating specific biological mechanisms underlying particular phenomena, and unifying different behaviours of the same system within a single framework (e.g. [27]). In contrast, simple algebraic (so-called normal forms) models provide deeper insights into the underlying dynamical processes at play, and whilst they lack the ability to prescribe specific biophysical mechanisms, they provide a window into unifying principles that exceed the confines of any one particular domain. A classic example is the logistic equation, which was derived as an abstraction of population dynamics [51] but became a mathematical paradigm for chaotic dynamics in complex systems. We presently show that a normal form of the Hopf bifurcation - a fifth order polynomial - is able to recapitulate the multistable and scale-invariant dynamics of human cortical activity when endowed with a subcritical instability and multiplicative noise. We also show how this model captures the dynamical principles at play in a detailed neural field model of cortical dynamics as well as a kinetic model of genetic regulation in a bacteriophage, hence suggesting universality in the biological world.

Regardless of their niche, biological systems share a number of competing constraints such as flexibility, robustness, cost efficiency, fidelity, and reproduction. Is it hence surprising that apparently diverse biological systems exploit similar dynamical mechanisms in addressing these? Given that universality underpins many dynamical systems observed in nature [52], and indeed normal form models are formally equivalent to broad classes of more complex systems under fairly general conditions, it is perhaps to be expected that simple models should capture fundamental dynamical processes that arise throughout biology. We have argued that the combination of a subcritical bifurcation with multiplicative noise satisfies two key constraints, namely spontaneous switching between multistable modes of activity (flexibility) and scale-invariant fluctuations between these modes over several orders of magnitude (fidelity). The presence of bistability in both the detailed neural field model [2] and the genetic regulatory system [3] was already known: here we suggest that they share the same dynamical mechanism. Although Hasty et al. [3] illustrated exemplar time series with both additive and parameter noise, we add specific predictions arising from a state-dependent stochastic term, namely that the fluctuations may be scale invariant and the dwell times in each mode will be long-tailed. This is consistent with effects documented in other genetic systems [48], possibly due to similar mechanisms.

By suggesting the functional form of the deterministic and stochastic terms in large-scale cortical dynamics, the present findings have direct and pragmatic implications for the analysis of neuroimaging data. Where stochastic processes have been previously introduced to dynamic models of cortical systems, they have almost invariably taken the form of a purely additive term. Our analysis mandates a state-dependent noise term for inversion of neural field (e.g., Jirsa and Haken [53], Robinson et al. [54]) and neural mass (e.g., Wendling et al. [55], David and Friston [56]) models from electrocortical data. We have given a proof-of-principle here by inverting the normal form model from two examples of bistable human EEG data (Figure 7). Naturally, formal inversion should leverage other (lower order) properties of the data such as spectral peaks and scaling regimes [25], [57] However, the bistable statistics are indispensable for disentangling the additive and multiplicative noise parameters, as well as the global properties of the system's phase space.

Moving beyond the exemplar model inversion method demonstrated here, a more formal framework, such as the variational framework implemented in Dynamic Causal Modeling (DCM) [58], would enable robust parameter estimation and model comparison. We provide a preliminary exploration of model space in duplets of parameters and show that (1) The space appears sufficiently well behaved (surfaces are smooth and unimodal), and (2) For fixed parameters, system statistics are Normally distributed. These observations suggest that the parameters will be identifiable and penalties for system complexity that rest upon the divergence between prior and posterior parameter distributions will be obtainable: Both of these are required for the variational model inversion scheme implemented in DCM [37]. This method has been successful in neuroscience for inverting both EEG and functional magnetic resonance imaging (fMRI) data. In fact the model typically used in DCM for fMRI is also a truncated power series (linear or 2nd-order), similar to our algebraic model, to which fluctuations [59] have been added. Our findings suggest that a higher order term (at least cubic) may be required for many of the rich behaviours observed near the edge of dynamical instabilities, and not necessarily only in EEG data. While EEG oscillations are much faster than fMRI responses, dwell time switching in cortical systems exhibits long tails and hence occurs well into the scales observable in fMRI data (∼10 sec). It is hence possible that a DCM with a higher order state term may be a better generative model for fMRI data than the current quadratic term. This is an empirical question that can be addressed using a suitable model inversion scheme [60], [61].

As noted before, a cubic polynomial is sufficient to describe the family of either sub- *or* supercritical Hopf bifurcations, but not both: A fifth order term is required to enable a smooth transition between the two [33]. Inclusion of higher order terms opens the possibility of modeling even more subtle features of the dynamics, such as sub-harmonics and nonlinear amplitude-frequency effects. These may be crucial to highly nonlinear neuronal computations such as pitch perception in the auditory system [62] and vibrotactile perception [63]. It is also important to note that the present normal form, which captures the basic dynamics of the biological systems we study, cannot be expected to capture all forms of multistability. In particular, the limit cycle is extinguished at the “lower end” of bistability (Figure 2A) through a saddle-node bifurcation. That is, the attractor loses structural stability and ceases to exist. An alternative dynamical scenario would be that such an attractor only loses asymptotic (Lyapunov) stability. That is, it remains an invariant set but loses attraction in one or more subspaces, hence becoming a saddle [64]. In this setting, system noise could still allow the system to itinerantly shadow the “ghost attractor” [4], although (in the absence of an attracting basin) the associated switching behavior may follow a simple Poisson process and not show any trapping (i.e. stretched, long-tailed dwell times). This dynamical scenario could only arise in a different normal form model.

Whilst we have argued that modeling state-dependent noise and higher order state terms in neurophysiological data has pragmatic implications, they also speak to fundamental computational processes in the brain. As recalled in the Introduction, state-dependent fluctuations are arguably a defining feature of perception (the “Weber-Fechner law”), movement (“Fitt's law”), and computation (“Hick's law”). Intriguingly, multistability co-exists with scale invariant fluctuations in these basic cognitive systems. For example, multistability is a well known property of the function (as exemplified by binocular rivalry) and physiology (see for example [65]) of the visual system. Likewise, Weber's law has well-known neurophysiological correlates in the early visual system [66]. Whilst the fixed ratio of the standard deviation with the mean intensity of a percept or motor action permits relative uncertainty to remain constant, the presence of multistability in these same systems additionally allows switching to distinct dynamical regimes which may confer adaptive advantage. If generative models that contain higher order terms are to provide stronger model evidence than simpler models (without those terms), it will arguably be most likely during experimental manipulations where such scale-free (or multistable) computations provide a performance advantage. That is, when the optimal generative model for the data embodies the same functional form as that required to optimize task performance.

## Materials and Methods

### Electroencephalographic data

Resting state EEG data were acquired from 16 healthy subjects using BrainAmp amplifiers (hardware bandpass filter, 0.1–250 Hz; BrainAmp; Brain Products) and EEG caps (Easy-Cap; FMS). Written informed consent was obtained from each subject prior to their participation. For detailed description of EEG data acquisition, preprocessing and analysis, please refer to [1], [2].

### Normal form model - numerical integration

Time series were obtained from numerical integration of (4) in the presence of stochastic fluctuations. We used Heun's integration scheme (an extension of the Euler integration into a two-stage second-order Runge–Kutta integration scheme [67]) with 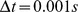.

In order to ensure stable and reproducible results, we obtained 10 different time series for each set of parameter values, starting from different initial conditions. Integration length was 900 s, matching the length of the EEG data sets. The first 10 seconds were always discarded to account for initial transients.

For all following analyses the amplitude time series *r(t)* was transformed to its Analytic signal 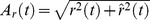
_,_ where 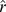 is the Hilbert transform of *r*, since the fitting of an exponential PDF, i.e. a chi-square distribution with two degrees of freedom (DoF), to a power time series mandates an underlying signal with two DoF. Substituting *r* by 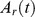 increases the DoF to two, thereby ensuring the valid application of the exponential PDF fitting procedure.

### Parameter estimates for bimodal PDFs

Power PDFs were obtained by partitioning the time series of 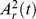 into 200 equally-sized bins and counting the number of observations in each bin. Using the maximum likelihood estimate (MLE), empirical PDFs were then fitted to two types of distribution functions: A simple exponential form [68] 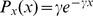, where x is the power and γ is the shape parameter; and a biexponential form 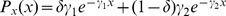, where *γ1* and *γ2* are the shape parameters of the two exponentials and δ is a weighting factor. We formally compared the bimodal to a unimodal fit using the Bayesian information criterion (BIC), which includes a penalty term for model complexity: 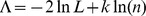, where L is the maximum likelihood estimate, i.e. the maximum value of the log likelihood function for the estimated model, n is the number of observations and k is the number of free parameters (*k = 1* for the unimodal fit and *k = 3* for the bimodal fit).Given two or more candidate models, the ‘best’ model will yield relatively low values of Λ – reflecting small residual variance after penalization for the number of free parameters. In order to gain a better insight into the functional form of the PDF, we formally evaluated the fitted PDFs in log-log coordinates.

### Parameter estimates for stretched exponential dwell time CDFs

Following estimation of bimodal exponential distribution functions, the respective dwell time distributions were characterized by estimating their cumulative distribution functions (CDFs) [28]. Using the same procedure as described in [1], [2] we fitted the stretched exponential form 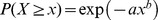 to the dwell time CDFs. In order to estimate the parameters *a* and *b*, the equation was rewritten as 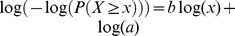. The parameters *a* and *b* were estimated from both the empirical and model data by means of a least squares linear regression in log(x)-log(log(P)) coordinates.

### Parametric analysis of noise and stability parameters

To map out the possible bistability and dwell time statistics, we explored the parameter space of our model by systematically varying stability parameters β and λ as well as the noise parameters η and ρ from (4). For λ>0, and 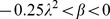 the system exhibits two attractors (a fixed point and a limit cycle) coexisting in parameter space, and hence expresses bistability, given an adequate noise term. We hence chose fixed values *λ* = 4, *β* = −3.4 and systematically varied the noise parameters *η* and *ρ* between 80 linearly equally spaced values in the defined ranges 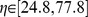, and 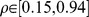 which were determined through iterative adjustment.

For each combination of values (80×80), we iterated (4) as described in section *Normal form model - numerical integration*. For each time series, we applied the same fitting procedures and parameter estimation methods as for the EEG data (as detailed above), yielding two key system statistics: the BIC difference, quantifying the relative goodness of fit of a bimodal versus a unimodal PDF, and the stretched exponential exponent *b* indicating the degree of stretching of the dwell time distributions for the low and high power modes. BIC difference and exponent *b* were taken as averages across the 10 calculated time series at each point.

The procedure was repeated for fixed noise parameters (*η* = 44.945 and *ρ* = 0.61) and varying stability parameters (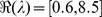,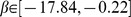) as well as for all remaining possible combinations of two fixed and to varying parameters.

### Corticothalamic neural field model

This computational model describes local mean field dynamics [36], [53], [69] of populations of excitatory and inhibitory neurons in the cortical gray matter as they interact with neurons in the specific and reticular nuclei of the thalamus. These dynamics are governed by physiologically derived nonlinear evolution equations that incorporate synaptic and dendritic dynamics, nonlinear firing responses, axonal delays, and synaptic gains between presynaptic impulses and postsynaptic potentials [25]. The activity within each neural population is described by three state variables - the mean soma membrane potentials *V_a_*(**x**, *t*) measured relative to resting, the mean firing rate at the cell soma *Q_a_*(**x**, *t*), and local presynaptic activity *φ_a_*(**x**, *t*) where the subscript *a* refers to the neural population (*e*: excitatory cortical; *i*: inhibitory cortical; *s*.: specific thalamic nucleus; *r*: thalamic reticular nucleus; *n*: nonspecific subcortical input). The global spatial mode is described by the eight first-order delay differential equations,
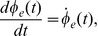
(m1)

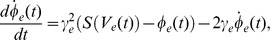
(m2)

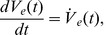
(m3)

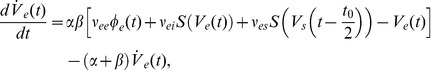
(m4)

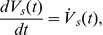
(m5)

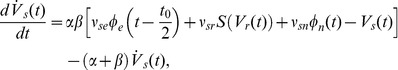
(m6)

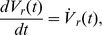
(m7)

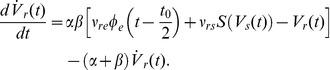
(m8) Note that equation (m6) contains the stochastic term
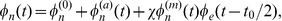
(m9) comprising additive 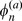 and multiplicative 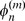 noise terms. The multiplicative term is modulated by the delayed corticothalamic feedback 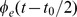.

Lengthy time series (4200 s) of the excitatory cortical presynaptic activity 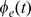 where used to represent the cortical sources of scalp EEG and obtained by numerical integration of the model. As for the normal form model, we used Heun's integration scheme with 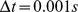.

## Supporting Information

Figure S1 Multiple (10,000) simulations of one fixed parameter setting reveal that each of the summary statistics shown in Figure 6 follows a normal distribution. A: Empirical probability distributions (blue) and fit of normal probability density function (red). B: Corresponding normal probability plots.
(TIF)
