# Supplementary figures and images for: A Canonical Model of Multistability and Scale-Invariance in Biological Systems
Source: PLoS Comput Biol. 2012 Aug 9;8(8):e1002634. doi: 10.1371/journal.pcbi.1002634 (PMC3415415; doi:10.1371/journal.pcbi.1002634)

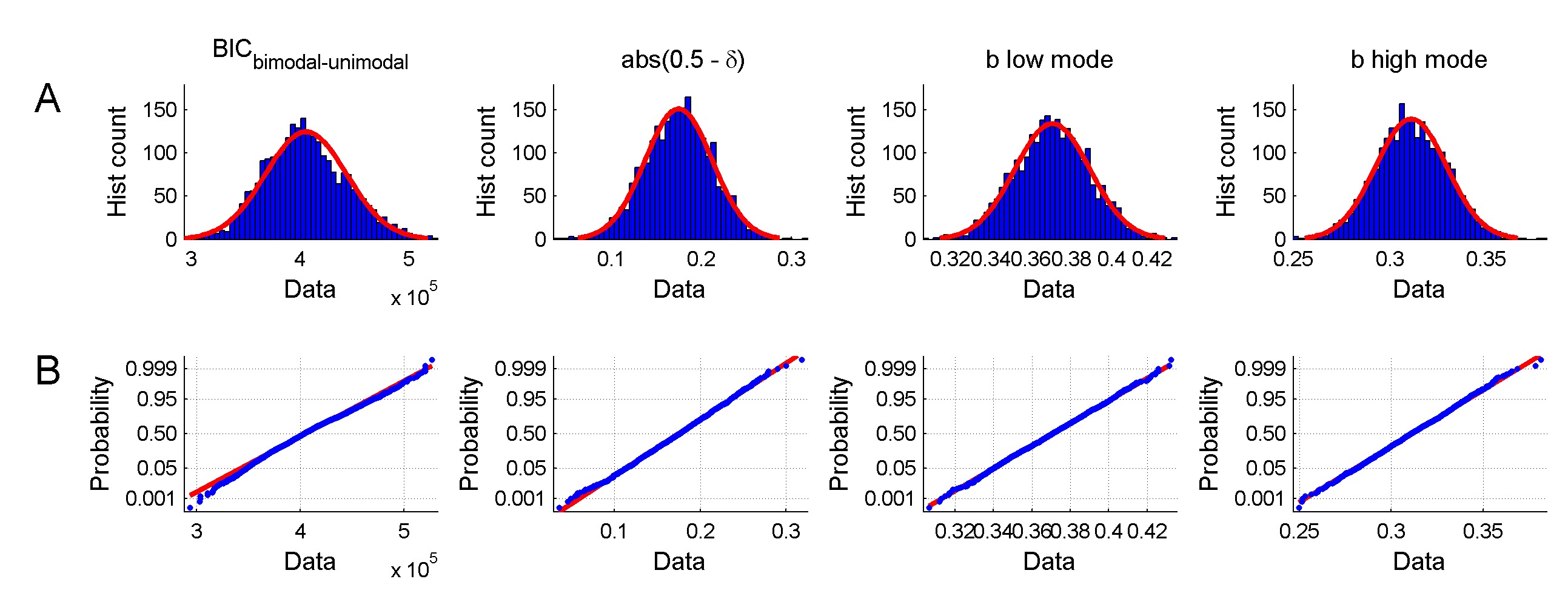

Supplement: Figure S1 — Multiple (10,000) simulations of one fixed parameter setting reveal that each of the summary statistics shown in Figure 6 follows a normal distribution. A: Empirical probability distributions (blue) and fit of normal probability density function (red). B: Corresponding normal probability plots. (TIF) [file pcbi.1002634.s001.tif]
